# OsMre11 Is Required for Mitosis during Rice Growth and Development

**DOI:** 10.3390/ijms22010169

**Published:** 2020-12-26

**Authors:** Miaomiao Shen, Yanshen Nie, Yueyue Chen, Xiufeng Zhang, Jie Zhao

**Affiliations:** State Key Laboratory of Hybrid Rice, College of Life Sciences, Wuhan University, Wuhan 430072, China; shenmm@whu.edu.cn (M.S.); nie_ys@whu.edu.cn (Y.N.); 2011202040109@whu.edu.cn (Y.C.); zhang1994@whu.edu.cn (X.Z.)

**Keywords:** *OsMre11*, mitotic cell cycle, DNA replication, DNA damage repair, root apical meristem

## Abstract

Meiotic recombination 11 (Mre11) is a relatively conserved nuclease in various species. Mre11 plays important roles in meiosis and DNA damage repair in yeast, humans and *Arabidopsis*, but little research has been done on mitotic DNA replication and repair in rice. Here, it was found that *Mre11* was an extensively expressed gene among the various tissues and organs of rice, and loss-of-function of *Mre11* resulted in severe defects of vegetative and reproductive growth, including dwarf plants, abnormally developed male and female gametes, and completely abortive seeds. The decreased number of cells in the apical meristem and the appearance of chromosomal fragments and bridges during the mitotic cell cycle in rice *mre11* mutant roots revealed an essential role of *OsMre11*. Further research showed that DNA replication was suppressed, and a large number of DNA strand breaks occurred during the mitotic cell cycle of rice *mre11* mutants. The expression of *OsMre11* was up-regulated with the treatment of hydroxyurea and methyl methanesulfonate. Moreover, OsMre11 could form a complex with OsRad50 and OsNbs1, and they might function together in non-homologous end joining and homologous recombination repair pathways. These results indicated that *OsMre11* plays vital roles in DNA replication and damage repair of the mitotic cell cycle, which ensure the development and fertility of rice by maintaining genome stability.

## 1. Introduction

DNA replication in the cell cycle and cell division is vital to the development and reproduction of all organisms. Complete and accurate DNA replication not only maintains the stability of the genome but also is important for accurate transmission of DNA genetic information to daughter cells. The replicon hypothesis related to DNA replication originated in the 1960s and was used to explain the control of initiation of bacterial replication [1]. In *E. coli*, DNA replication is controlled by the starting gene *oriC*. After the initiation of replication, hemimethylated *oriC* can be generated, and other proteins can bind to it and prevent the assembly of new replication initiation complexes [1,2]. In eukaryotes, DNA replication begins in the S phase of the cell cycle [3]. When the recognition complex binds to an initiation site, replication begins. The proteins CDC6 (cell division cycle 6) and CDT1 (chromatin license and DNA replication factor 1) participate together in DNA replication initiation, and MCM (recruit minichromosome maintenance) complex facilitates DNA replication [3,4]. When DNA replication is wrong or blocked, certain proteins are involved in repairing the defective DNA, and then, the replication event continues. However, when the loss-of-function occurs in factors related to DNA replication, it may cause a large number of genomic instability and affect the normal growth and development of organisms [5].

Organisms in nature are often affected by endogenous and exogenous factors, resulting in DNA double-strand breaks (DSBs). Endogenous DNA damage is often generated in the process of DNA replication and transposon excision, while exogenous DNA damage tends to be induced by ionizing radiation, biochemical reagents, ultraviolet irradiation, and so on [6]. DSBs are highly toxic lesions that can cause chromosomal translocations and rearrangements that ultimately lead to senescence, cell death, or tumorigenesis [7]. Therefore, maintaining genome integrity is vital for precise inheritance of genes and cell survival. 

Once DSBs are produced, specific proteins are recruited to repair them. There are two major DSB repair pathways, non-homologous end joining (NHEJ) and homologous recombination (HR) [8,9]. NHEJ is the main pathway of DSB repair and runs through the entire life cycle. In this pathway, the two ends of the broken DNA can connect together quickly and directly using little or no homologous sequence, resulting in genetic mutation or chromosomal aberration. HR is a more accurate repair pathway compared with NHEJ. In this pathway, the broken DNA end uses sister chromatin or a homologous sequence as a template to synthesize new DNA, which only works in the S and G2 phases of the cell cycle [10]. 

Meiotic recombination 11 (Mre11), radiation sensitive 50 (Rad50), and Nijmegen breakage syndrome 1 (Nbs1) (or X-ray sensitive 2 [Xrs2] in *Saccharomyces cerevisiae*) form the MRN (or MRX) complex. In the presence of damage, MRN is the first sensor responding to DSBs and is critical for recruiting other repair proteins and activating ATM (Ataxia telangiectasia mutated) and ATR (ATM and Rad3 related) kinases [11,12,13]. Mre11 and Rad50 are highly conserved in biological evolution, while Nbs1/Xrs2 is less conserved and only found in eukaryotes [14]. The MRN complex can recognize DSB ends. Once it binds to DNA molecules, the DNA unwinds to facilitate the successful repair [15]. The gene *Mre11* encodes a nuclease protein that was first identified by screening in yeast. Mre11 is the critical protein in the MRN complex and digests the DNA by its 3′ to 5′ exonuclease activity and 5′ to 3′ endonuclease activity [16,17]. Rad50 is an ATPase and has two long coiled-coil domains, acting as an intermediate bridge to connect the broken DNA end [18]. Nbs1 is an important component in determining the location of DNA damage, controlling the activity of the MRN complex, detecting DNA damage signaling, and recruiting ATM to DSB ends [19,20,21].

In the S phase and G2 phase of the cell cycle, Mre11 cleaves the 5′-strand of the DNA DSB ends to produce a single-stranded DNA gap through its endonuclease activity [22,23]. During this process, the NHEJ pathway is prevented, DNA2 (DNA replication helicase/nuclease 2) and EXO1 (Exonuclease 1) proteins bind the DNA to cut for a longer distance, which is important for single-strand DNA invasion, homologous recombination, and activation of ATR [17,24,25]. The activation of ATM depends on the interaction between Mre11 and Nbs1. ATM phosphorylates many repair proteins, including H2AX, Mre11, Nbs1, Sae2/CtIP (SUMO-activating enzyme 2/CTBP-interacting protein), CHK1 (checkpoint kinase 1), CHK2 (checkpoint kinase 2), and ATM itself, leading to cell cycle arrest and DNA repair pathway activation [26,27]. 

Mutation of *Mre11* or *Rad50* in *Arabidopsis* led to abortion of siliques and showed high sensitivity to damage agents. In the two mutants, ATR, but not ATM, phosphorylates the histone H2AX, and the ATR-dependent phosphorylation is accumulated extensively in the S-phase nucleus [12,28,29]. Moreover, the mutation of the *Mre11* gene leads to loss of some chromosomes and DNA repair ability, and increases the sensitivity to damage agents [30,31]. In *Caenorhabditis elegans*, the *Mre11* gene plays an important role in meiosis. There is no crossing-over formation during meiosis of the *mre11* mutant, while the synapsis is normal [32]. In mouse, loss of Mre11 nuclease activity increases genomic instability and early embryonic lethality [33]. In humans, the *mre11* mutant gives rise to ataxia-telangiectasia-like disorder [34]. In rice, the homolog of *Mre11* has been identified and functions in meiotic progression. When *OsMre11* is not expressed, neither homologous synapsis nor the end of DNA processing can push forward normally [35]. Nonsense mutations in the *Nbs1* gene are fatal to mammals, because Nbs1 cannot mediate DNA-damage signaling and recruit ATM to the damage sites for precise repair [36]. In humans, Nbs1 is considered to be the genetic basis of Nijmegen breakage syndrome (NBS) which is a rare disease connected to chromosomal instability. The clinical feature of the disease is irradiation sensitivity, cancer susceptibility, immunodeficiency, short stature, microcephaly, and intellectual disability [34]. 

This work provides new insight into future investigations aimed at understanding the molecular mechanisms of *Mre11* in DNA replication and damage repair during the mitosis of rice.

## 2. Results

### 2.1. Mre11 Is Conserved in Various Species and Expressed Extensively during the Growth and Development of Rice

To investigate the conservation of the Mre11 protein among various species, the full-length amino acid sequences of OsMre11 and its orthologs were subjected to sequence comparison (Figure S1). The result showed that OsMre11 and its orthologs exhibit a relatively conserved amino acid sequence. By analyzing the structure of OsMre11 protein (http://pfam.xfam.org/search#tabview=tab1), we found that it contains two functional domains, metallophos phosphoesterase and DNA-binding domains (Figure S1). Further phylogenetic analysis showed that Mre11 proteins were widespread in prokaryotes, animals, and higher plants (Figure S2).

**Figure 1:**
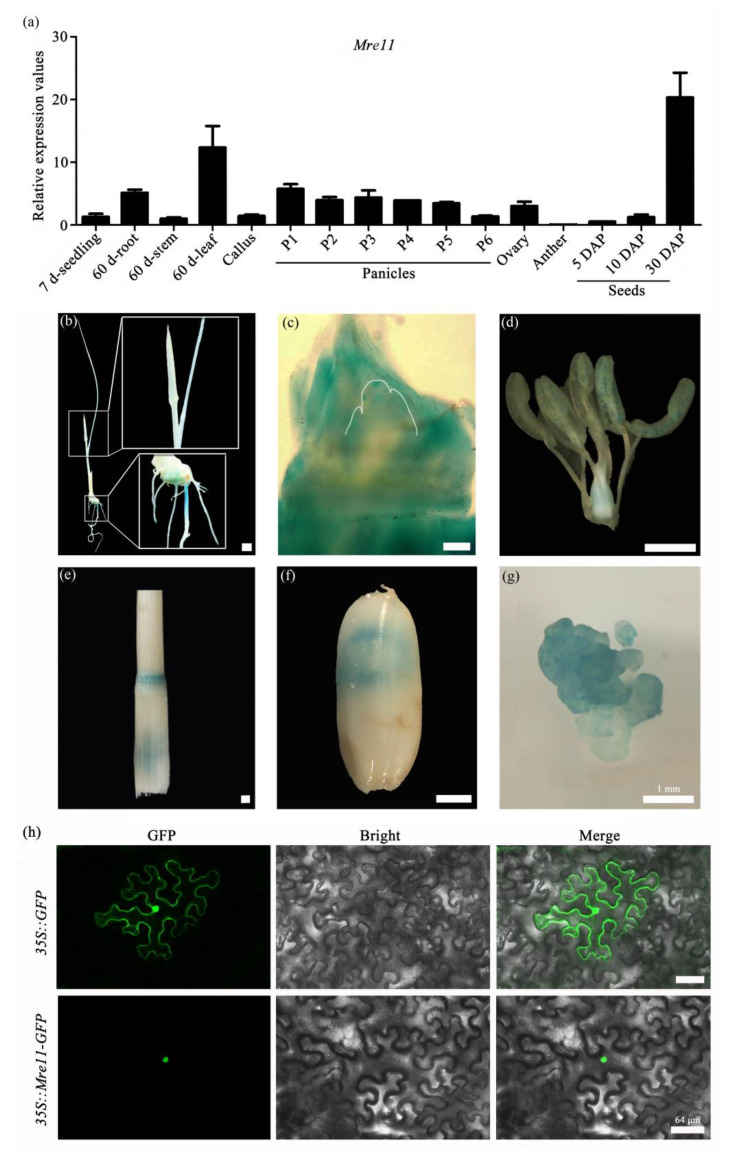
Expression pattern of *OsMre11* gene in rice. Expression pattern of *OsMre11* gene in rice. (**a**) Expression level of *OsMre11* gene in 7-day-old seedling, 60-day-old root, 60-day-old stem, 60-day-old leaf, callus, panicles (P1: 0–3 cm spikelet; P2: 3–5 cm spikelet; P3: 5–10 cm spikelet; P4: 10–15 cm spikelet; P5: 15–22cm spikelet; P6: 22-30cm spikelet), ovary, anther, and seeds (5DAP: 5 days after pollination; 10DAP: 10 days after pollination; 30DAP: 30 days after pollination). (**b**–**g**) GUS (β-Galactosidase) expression pattern in different tissues and organs. GUS expression in 7-day-old seedling after germination (**b**), shoot apical meristem (**c**), pistil and stamen (**d**), 60-day-old stem (**e**), mature seed (**f**), transgenic positive callus (**g**). (**b**,**d**–**g**) Scale bars represent 1 mm. (**c**) Scale bar represents 0.1 mm. (**h**) Subcellular localization of OsMre11 in tobacco epidermal cells. The 35S::GFP was used as a negative control. Scale bars represent 64 µm.

To investigate the temporal and spatial expression pattern of *OsMre11*, we detected the transcriptional level of *OsMre11* in different vegetative and reproductive tissues via qRT-PCR assay and found that *OsMre11* was widely expressed in all examined tissues, with relatively high expression values in 60 d leaves and 30DAP seeds (Figure 1a). Furthermore, the *OsMre11*-driven tissue-specific GUS (β-Galactosidase) expression analysis showed that *OsMre11* was expressed in young leaf, root and root tip (Figure 1b), shoot apical meristem (SAM) (Figure 1c), pistil, stamen (Figure 1d), 60-day-old stem (Figure 1e), mature seed (Figure 1f), and callus (Figure 1g). To investigate the subcellular localization of OsMre11 protein, the *35S::Mre11-GFP* vector was constructed and transferred into tobacco leaf epidermis. Fluorescence observation showed that the GFP signals were specifically localized in the nuclei (Figure 1h). These results suggest that OsMre11 is a conserved nucleoprotein that may have vital functions in multiple developmental stages of rice.

### 2.2. Knock-Out of OsMre11 Causes Dwarfism of the Rice Plant

**Figure 2:**
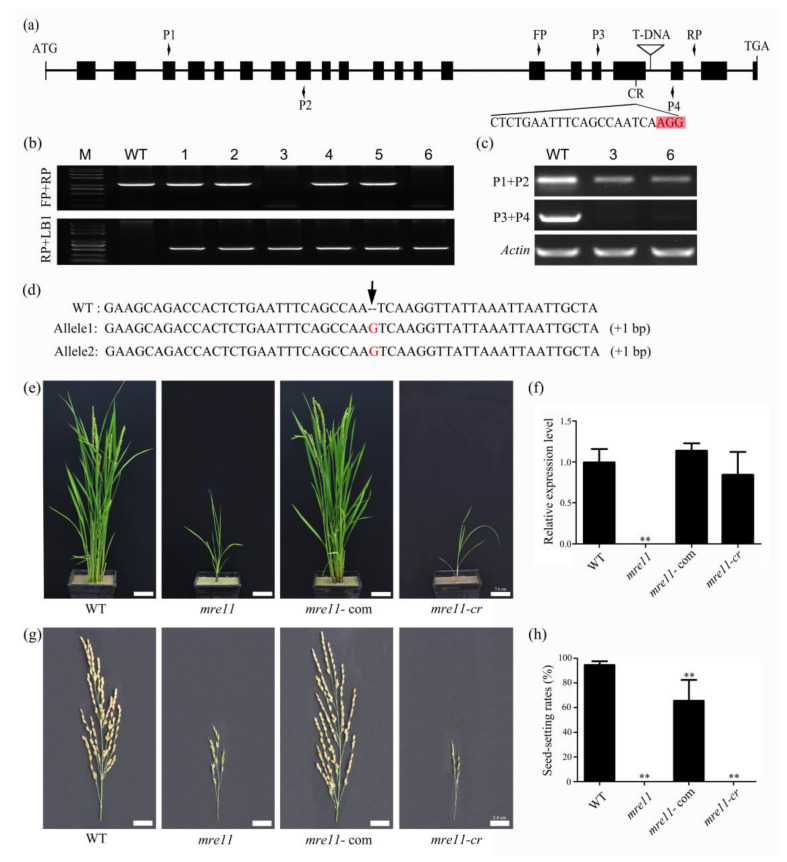
Identification and phenotypic characteristics of the *mre11* mutant in rice. Identification and phenotypic characteristics of the *mre11* mutant in rice. (**a**) Schematic diagram of the *Mre11* gene in rice shows the positions of T-DNA insertion and CRISPR/Cas9 (clustered regularly interspaced short palindromic repeats/Cas9) editing site. Exons are depicted as black box; while introns are depicted as lines. Red rectangle highlights the CRISPR/Cas9 editing site protospacer adjacent motif sequence. (**b**) Identification of the homozygous and heterozygous *mre11* mutants by assay method of three primers. (**c**) Semiquantitative analysis of *Mre11* gene in the number 3 and 6 of rice plants. (**d**) Sequence analysis of *Mre11* mutation site in the *mre11-cr* plant. An arrow indicates the location of genetic editing site, inserting a base G. (**e**) Phenotypes of wild-type plant (WT), *mre11* mutant plant, complemented plant (*mre11*-com), and CRISPR/Cas9 transgenic plant (*mre11-cr*). Scale bars represent 7.6 cm. (**f**) Relative expression level of *OsMre11* gene in WT, *mre11*, *mre11*-com, and *mre11-cr* plants. (**g**) Panicle phenotypes of WT, *mre11*, *mre11*-com, and *mre11-cr* plants. Scale bars represent 2.4 cm. (**h**) The average seed-setting rates of each panicle in WT, *mre11*, *mre11*-com, and *mre11-cr* plants. The numbers of panicles are more than 24. The two asterisks represent a statistically significant difference according to Student’s t-test (**, *p* < 0.01).

To investigate the function of *OsMre11* in rice, the T-DNA mutant was first identified, and the primers were designed to identify the precise mutation position. The result showed that the T-DNA insertion site of the *mre11* was located in the 19th intron of *OsMre11* (Figure 2a). Through genotypic identification in the progenies, we obtained wild type (WT), heterozygous (HZ), and homozygous (HM) plants (Figure 2b) and found that there was no *OsMre11* expression near T-DNA insertion in the 3rd and 6th homozygous plants by semi-quantitative PCR (Polymerase Chain Reaction) technique (Figure 2c). Furthermore, the phenotype characteristics of all genotypes were observed, and no developmental defects appeared in WT or heterozygote plants during vegetative growth, whereas the homozygous plants of the *mre11* mutant were dwarf (Figure 2e). To verify that the dwarf phenotype was caused by the mutation of *Mre11* in rice, the complementation assay was performed by transforming the full-length genomic fragment of *OsMre11* into the *mre11* callus, forming transgenic plants. We found that vegetative growth and gene expression of the complementary plants (*mre11*-com) returned to normal levels (Figure 2e,f). Simultaneously, the CRISPR/Cas9 (clustered regularly interspaced short palindromic repeats/Cas9) system was used to create another mutant *mre11-cr*. In this mutant, the editing site was located in the 19th exon close to the T-DNA (Figure 2a), and sequencing of PCR amplified *OsMre11* genomic DNA from rice transgenic positive plants showed that each chain of DNA inserted a guanine base, indicating it was a homozygous mutant (*mre11-cr*) (Figure 2d). Although the *OsMre11* expression level in the *mre11-cr* mutant was close to rice WT and the complementary plants (Figure 2f), the phenotype analysis showed that the development characteristics of *mre11-cr* were similar to the T-DNA *mre11* mutant (Figure 2e,g). Because *mre11-cr* had a point mutation, the mutant had no effect on its gene expression. These results indicate that *OsMre11* is essential for normal vegetative growth and development in rice.

**Figure 3:**
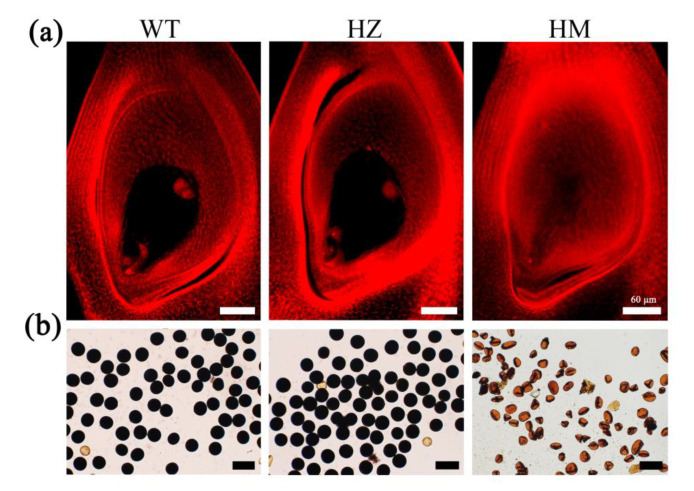
Phenotype characteristics of male and female gametophytes in rice heterozygous and homozygous mutants. Phenotype characteristics of male and female gametophytes in rice heterozygous and homozygous mutants. (**a**) Pictures of ovary transparency, displaying defective embryo sac in homozygous mutants (HM) compared with wild type (WT) and heterozygous (HZ). (**b**) Pictures of pollens stained with KI-I_2_, showing abortion pollens in HM mutants compared with WT and HZ. Scale bars represent 60 µm.

Moreover, the seed-setting rates were up to 94.61% in the WT plants and 65.55% in the *mre11*-com T_2_ generation plants, while the seeds of the *mre11* and *mre11-cr* mutants were completely aborted (Figure 2h,g). The embryo sac transparency and KI-I_2_ staining showed that the female and male gametophytes of HZ plants had no obvious abnormality, but those of *mre11* (HM) were completely aberrant, leading to the aborted seeds (Figure 3a,b). These results suggest that *OsMre11* plays a crucial role in the development of female and male gametophytes.

For the other two components of the MRN complex, the expression of *OsRad50* and *OsNbs1* was extensive in the examined tissues and organs (Figure S3a,b), indicating that they are extensively expressed genes similar to *OsMre11*. These results suggest that Rad50 and Nbs1 may play roles in the growth and development of rice together with Mre11.

### 2.3. The Cell Division Activity in the Root Apical Meristem of the mre11 Mutant Was Inhibited

**Figure 4:**
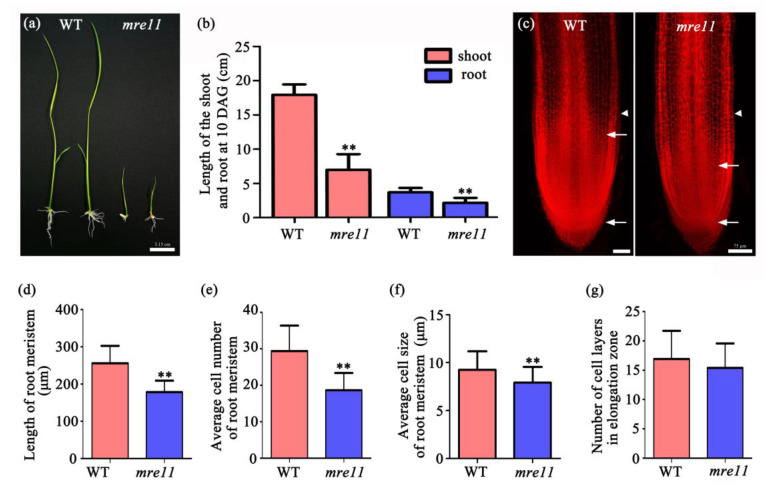
Characteristics of roots in WT (Wild-type) and the *mre11* mutant of rice. Characteristics of roots in WT and the *mre11* mutant of rice. (**a**) The phenotypes of 10-day-old WT and the *mre11* seedlings. Scale bar represents 3.15 cm. (**b**) Shoot (red) and root (blue) length of 10-day-old WT and the *mre11*. The numbers of WT and the *mre11* were 40 and 14, respectively. (**c**) Phenotype characteristics of root apical meristem in the WT and the *mre11* of rice. The two arrows indicated the areas of root apical meristem. The two arrowhead in each root showed the location of counting cell layers from outside to inside in elongation zone of the root tip. Scale bars represent 75 µm. (**d**) The analysis in length of root apical meristem. (**e**) Average cell number of root apical meristem. (**f**) Average cell size of root apical meristem. (**g**) Number of cell layers from outside to inside in root elongation zone. (**d**–**g**) The numbers of WT and the *mre11* are 37 and 31, respectively. The two asterisks represent a statistically significant difference according to Student’s t-test (**, *p* < 0.01).

The shoots and roots of the *mre11* seedlings that germinated for 10 days were significantly shorter than those of the WT seedlings (Figure 4a). The average length of shoots and roots was 61.18% and 42.29% shorter than the WT seedlings, respectively (Figure 4b). To further observe the cell size and number of the root apical meristem (RAM), we performed the root tip transparent and staining assay in 10-day-old seedlings (Figure 4c), and found that the RAM of the *mre11* mutant was 30.36% shorter in length (Figure 4d), 36.75% less in cell number (Figure 4e), and 14.39% smaller in cell size (Figure 4f) than the WT seedlings. However, there was no difference in the number of cell layers in the elongation zone (Figure 4g). These results imply that the cell division activity in the *mre11* mutant is inhibited.

### 2.4. Mitotic Division Is Aberrant in the Root Apical Meristem of the mre11 Seedlings

**Figure 5:**
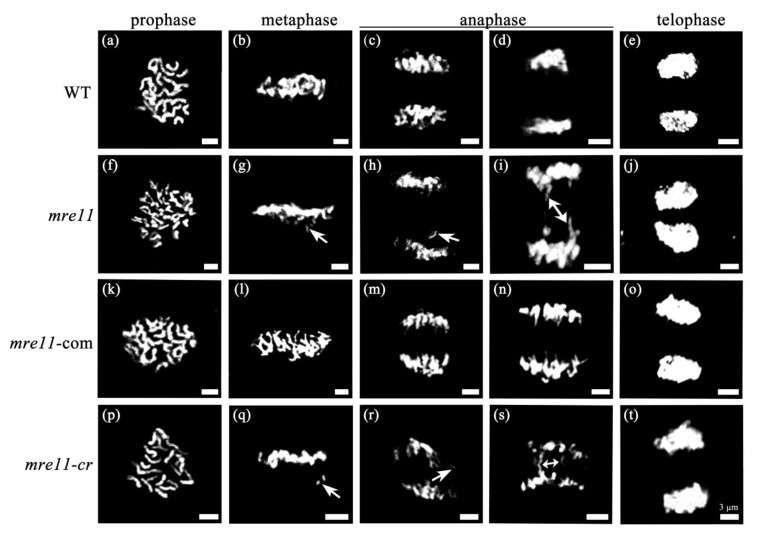
Chromosomal behavior of mitosis in WT and the mutants of rice. Chromosomal behavior of mitosis in WT and the mutants of rice. The root tip cells were detected in 7DAG seedlings of WT, *mre11*, *mre11*-com, and *mre11-cr*. (**a**–**e**) Chromosomal behavioral characteristics of prophase, metaphase, anaphase, and telophase in WT. (**f**–**j**) Chromosomal behavioral characteristics of prophase, metaphase, anaphase, and telophase in the *mre11*. (**k**–**o**) Chromosomal behavioral characteristics of prophase, metaphase, anaphase, and telophase in the *mre11*-com. (**p**–**t**) Chromosomal behavioral characteristics of prophase, metaphase, anaphase, and telophase in the *mre11-cr*. (**g**,**h**,**q**,**r**) One sided arrows showed lagging chromosomes during metaphase and anaphase. (**i**,**s**) Double sided arrows showed chromosome bridges during anaphase. Scale bars represent 3 µm.

To investigate the defect in cell division in the *mre11* mutant, the mitotic chromosome morphology and segregation in the root tip cells were analyzed. As shown in Figure 5, chromatin was condensed to form chromosomes in prophase (Figure 5a,f,k,p), and the chromosomes of WT and *mre11*-com were regularly arranged on the equatorial plate in metaphase (Figure 5b,l), while there were many chromosomal fragments near the equatorial plates in the *mre11* and *mre11-cr* mutants (Figure 5g,q). In anaphase, sister chromatids of chromosomes moved to the two poles of cells, while some cells contained lagging chromosomal fragments and chromosomal bridges in the *mre11* and *mre11-cr* but not in WT and *mre11*-com (Figure 5c,d,h,i,m,n,r,s). In telophase, two daughter cells were generated (Figure 5e,j,o,t). The chromosome abnormal ratio reached up to 25.86% (*n* = 58) in the *mre11* and 34.78% (*n* = 23) in the *mre11-cr* seedlings, while there was no abnormities in WT (*n* = 76) and *mre11*-com (*n* = 41) seedlings. The results indicate that *OsMre11* is required for ensuring well-balanced mitosis in rice growth and development.

### 2.5. OsMre11 Functions in DNA Replication during Mitosis

**Figure 6:**
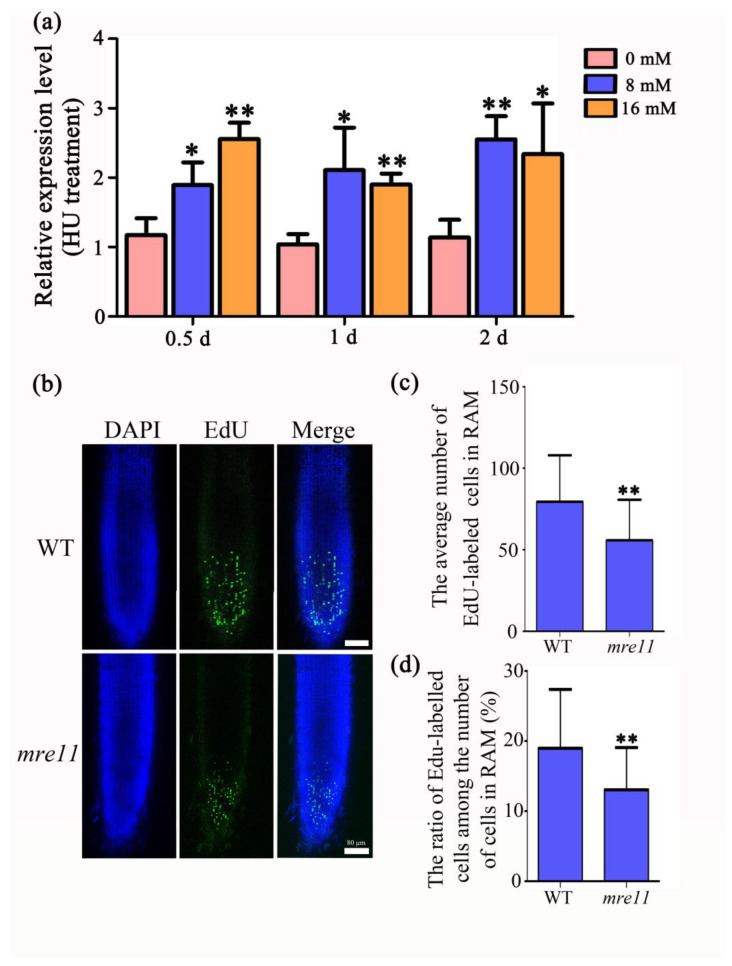
*OsMre11* is involved in DNA replication of rice root. *OsMre11* is involved in DNA replication of rice root. (**a**) Relative expression level of *Mre11* with the treatment of HU. The concentration of HU is 0, 8, and 16 mM. The materials were collected after the treatment of 0.5, 1, and 2 days. (**b**) Signals of ethynyl deoxyuridine (EdU) staining in 7DAG roots of rice WT and *mre11* seedlings. DAPI is 4ʹ, 6-diamidino-2- phenylindole dihydrochloride. Scale bars represent 80 µm. (**c**) The average number of EdU-labeled cells in root apical meristem. (**d**) The ratio of EdU-labeled cells among the number of cells in root apical meristem. The numbers of WT and the *mre11* are 24 and 18, respectively. The asterisks represent a statistically significant difference according to Student’s *t*-test (*, *p* < 0.05; **, *p* < 0.01).

A single cell cycle includes two stages, interphase and a mitotic division phase. Interphase is an active material preparation stage for mitosis, completing the replication of DNA molecules and the synthesis of related proteins. Interphase is divided into G1, S, and G2 phases, and DNA replication is mainly in S phase. To elucidate the role of *OsMre11* in the cell cycle, DNA replication inhibitors hydroxyurea (HU) and aphidicolin were applied to treat the 10 DAG seedlings. The results showed that the expression of *OsMre11* increased with the HU treatment (Figure 6a), but no obvious change was observed with the aphidicolin treatment (Figure S4a,b). Ethynyl deoxyuridine (EdU), an analog of thymidine, can be incorporated into chromosomes during DNA replication, and it is often used to label S-phase cells [37]. A staining assay with EdU showed that there was an obviously lower proportion of EdU-labeled cells in the RAM of *mre11* than those in WT (Figure 6b,c), and the ratio of EdU-labeled cells among the number of cells in RAM of the *mre11* was significantly lower than that of WT (Figure 6d), indicating that S-phase DNA replication is impaired in the *mre11* seedlings. Therefore, *OsMre11* is a critical factor for mitotic DNA replication in rice.

### 2.6. OsMre11 Is Essential for DNA Damage Repair in Somatic Cells

**Figure 7:**
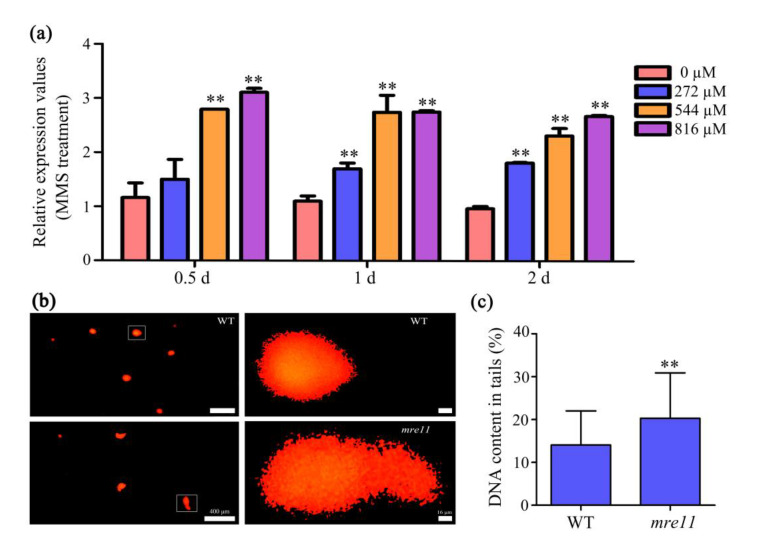
*OsMre11* is involved in DNA damage repair of rice. *OsMre11* is involved in DNA damage repair of rice. (**a**) Relative expression level of *OsMre11* in the 10DAG seedlings with the treatment of MMS reagent. The concentration of MMS was 0, 272, 544, and 816 µM, respectively. Materials were collected after the treatment of 0.5, 1, and 2 days. (**b**) Pictures of comet assay in the 10DAG seedling roots of WT and *mre11*. The images on the right are magnified images in the white boxes on the left. Scale bars represent 400 µm on the left and 16 µm on the right. (**c**) The DNA contents of the tail of nuclei in root cells of WT and the *mre11* seedlings. The numbers of WT and the *mre11* are 82 and 76, respectively. The two asterisks represent a statistically significant difference according to Student’s t-test (**, *p* < 0.01).

To prove whether the mutation of *OsMre11* was involved in the DNA damage response, the WT seedlings were treated with methyl methanesulfonate (MMS) and mitomycin C (MMC) to detect the expression level of *OsMre11*. MMS and MMC are both DNA damage reagents. MMS can only cause double strand breaks, while MMC can cause single strand breaks or double strand breaks. The results showed that the expression of *OsMre11* was up-regulated after treatment with the MMS reagent (Figure 7a), but there was no change after treatment with the MMC reagent (Figure S4c,d), suggesting that *OsMre11* has a higher sensitivity to MMS and may be involved in DSB repair. To verify this finding, we conducted a comet assay, also known as single-cell gel electrophoresis, to evaluate the strength of genotoxicity by detecting the degree of DNA strand damage (Figure 7b). The comet assay can effectively detect and quantify the extent of DNA single- and double-strand breakage in cells [38]. The more severe the DNA was damaged, the more fragments were produced, the more DNA breaks migrated under the same electrophoresis conditions, and the longer the migration distance. CometScore software calculation showed that DNA content in tails of the *mre11* mutant was nearly 1.44 times of WT (Figure 7c), indicating that *OsMre11* plays an important role in DNA DSB repair.

### 2.7. OsMre11 Plays Crucial Roles through Interacting with OsRad50 and OsNbs1

To further study the conservation of OsMre11 at the secondary structure level, we constructed the three-dimensional structure in different species based on the homolog CtMre11 in *Chaetomium thermophilum* (Figure S5). The Mre11 proteins exhibited a high degree of similarity in the secondary structure and can form a dimer with itself. In humans, yeast, and *Arabidopsis*, Mre11 can interact with Rad50 and Nbs1 to play biological roles together [12,39,40], but it is not clear whether Mre11 can interact with Rad50 and Nbs1 in rice. Therefore, we constructed the secondary structure of Rad50 and Nbs1 (Figure S5) and found that they were similar and conserved in various species. Rad50 could also form a dimer by itself, but Nbs1 could not. 

**Figure 8:**
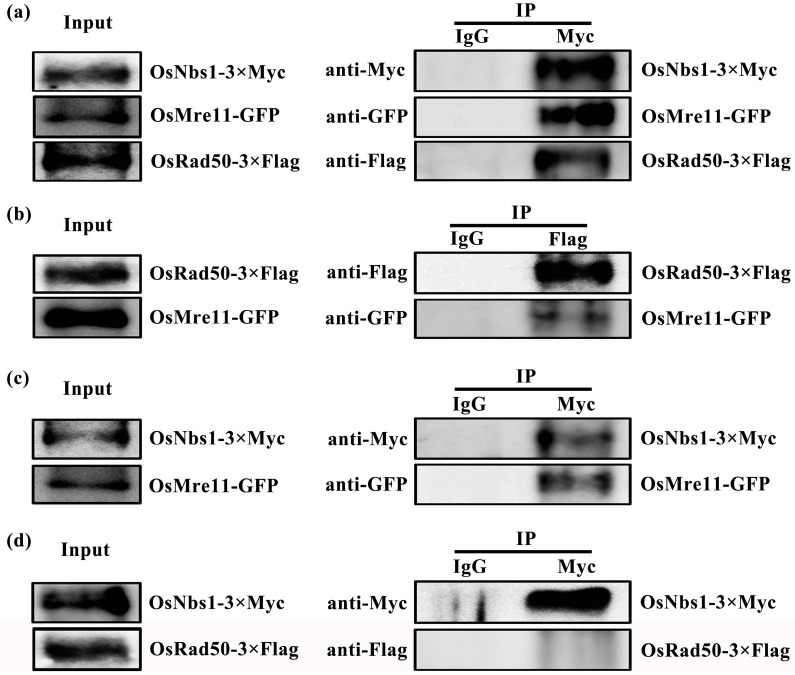
The interaction relationship between OsMre11, OsRad50, and OsNbs1 by coimmunoprecipitation (Co-IP) assay. The interaction relationship between OsMre11, OsRad50, and OsNbs1 by coimmunoprecipitation (Co-IP) assay. (**a**) The 35S::OsNbs1-3×Myc, 35S::OsMre11-GFP, and 35S::OsRad50-3×Flag were immunoprecipitated using anti-Myc antibody, and analyzed using anti-Myc, anti-GFP, and anti-Flag antibodies. (**b**) The 35S::OsRad50-3×Flag and 35S::OsMre11-GFP were immunoprecipitated using anti-Flag antibody, and analyzed using anti-Flag and anti-GFP antibodies. (**c**) The 35S::OsNbs1-3×Myc and 35S::OsMre11-GFP were immunoprecipitated using anti-Myc antibody, and analyzed using anti-Myc and anti-GFP antibodies. (**d**) The 35S::OsNbs1-3×Myc and 35S::OsRad50-3×Flag were immunoprecipitated using anti-Myc antibody, and analyzed using anti-Myc and anti-Flag antibodies. (**a**–**d**) IgG was the negative control group.

Subsequently, we performed a bimolecular fluorescence complementation (BiFC) assay in tobacco leaves using a transient transformation technique and detected that the YFP signals in transformed leaf cells co-expressing the constructs OsMre11-YN and OsMre11-YC, OsRad50-YN and OsRad50-YC, OsMre11-YN and OsRad50-YC, OsMre11-YN and OsNbs1-YC, OsRad50-YN and OsMre11-YC, and OsNbs1-YN and OsMre11-YC (Figure S6). The results showed that OsMre11 and OsRad50 can interact with themselves (Figure S6b,d), but OsNbs1 cannot (Figure S6f). OsMre11 can interact with OsRad50 and OsNbs1 (Figure S6g–i,k), but OsRad50 cannot interact with OsNbs1 (Figure S6j,l). To further verify the interactive relationship between OsMre11, OsRad50, and OsNbs1, coimmunoprecipitation tests and analysis were conducted (Figure 8). When the three fused proteins, OsMre11-GFP, OsRad50-3×Flag, and OsNbs1-3×Myc were transiently co-expressed in tobacco leaf cells, the OsMre11-GFP and OsRad50-3×Flag fusion proteins could be coimmunoprecipitated with OsNbs1-3×Myc (Figure 8a). When two fused proteins, OsMre11-GFP and OsRad50-3×Flag were transiently co-expressed in tobacco leaf cells, OsMre11-GFP could be coimmunoprecipitated with OsRad50-3×Flag (Figure 8b). When OsMre11-GFP and OsNbs1-3×Myc were transiently co-expressed in tobacco leaf cells, OsMre11-GFP could be coimmunoprecipitated with OsNbs1-3×Myc (Figure 8c). However, when OsRad50-3×Flag and OsNbs1-3×Myc were transiently co-expressed in tobacco leaf cells, OsRad50-3×Flag could not be coimmunoprecipitated with OsNbs1-3×Myc (Figure 8d). Therefore, the results suggest that OsMre11 may act as an intermediate bridge to connect OsRad50 and OsNbs1, forming the Rad50-Mre11-Nbs1 complex to function together in rice growth and development.

### 2.8. Transcriptome Comparative Analysis of Rice Wild Type and the mre11 Mutant

**Figure 9:**
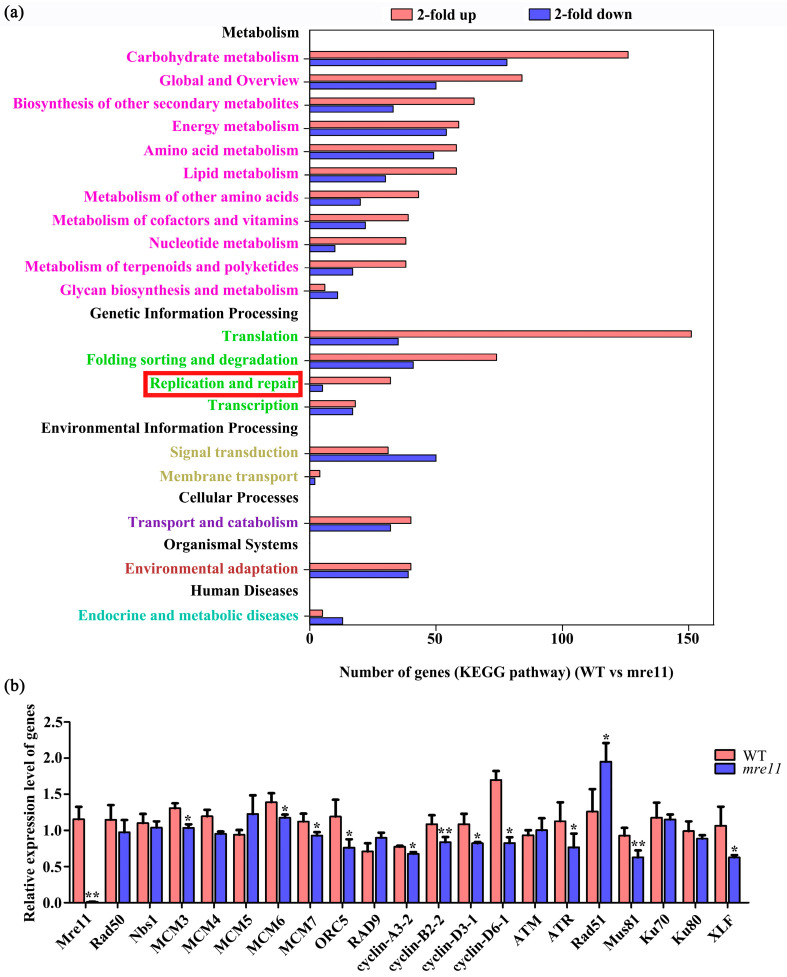
RNA sequencing analysis and verification of gene expression in rice WT and the *mre11* seedlings. RNA sequencing analysis and verification of gene expression in rice WT and the *mre11* seedlings. (**a**) The number of 2-fold up and down-regulated genes in the Kyoto Encyclopedia of Genes and Genomes (KEGG) pathway (WT vs. *mre11*). Many genes related to replication and repair had changed significantly in the *mre11* compared with WT (in the red frame). (**b**) The relative expression level of genes related to DNA replication, cell cycle, and DNA repair in WT and the *mre11* seedlings. The asterisks represent a statistically significant difference according to Student’s t-test (*, 0.01< *p* <0.05; **, *p* < 0.01).

To further research the effects of *OsMre11* mutation on gene expression, a transcriptome analysis of WT and the *mre11* seedlings was performed. The column chart of differentially expressed genes, at least a two-fold difference in expression level, showed that 2978 genes were up-regulated and 2396 genes were down-regulated (Figure S7a) in the *mre11* mutant compared with WT. Classification analysis of gene ontology (GO) based on RNA-seq data showed that the majority of the genes in the *mre11* mutant involved in biological process, molecular function, and cellular component were up-regulated (Figure S7b). Further observation on the number of genes in the Kyoto Encyclopedia of Genes and Genomes (KEGG) pathway showed that the expression of many genes related to replication and repair had changed significantly in the *mre11* compared with WT, indicating that the DNA replication and repair pathways were activated to some extent (Figure 9a). Among the KEGG pathway results, the expression of some genes involved in homologous recombination repair pathway and non-homologous end joining repair pathway were changed, such as *Mre11*, *RPA*, *BRCA2*, *Rad51*, *Rad54*, *ploδ*, *BLM*, *Mus81*, and *XLF* [41,42,43,44]. These results indicate that OsMre11 may function in the cell biological process, including cell division, DNA replication, and damage repair. To further verify the possibility, we performed the qRT-PCR experiment and found that the expression level of 12 genes related to DNA replication (*MCM3*, *MCM6*, *MCM7*, and *ORC5*), cell cycle (*cyclin-A3-2*, *cyclin-B2-2*, *cyclin-D3-1*, and *cyclin-D6-1*), and DNA repair (*ATR*, *Rad51*, *Mus81*, and *XLF*) changed significantly in the *mre11* compared with WT (Figure 9b), indicating *OsMre11* may be involved in the events of DNA replication, cell cycle, and DNA damage repair.

## 3. Discussion

### 3.1. Mre11 Is Indispensable for Growth and Development 

Mre11 is a nuclease and central component of the MRN complex. When DNA is damaged due to various factors, Mre11 can bind to the damaged DNA ends and excise them [17]. When DNA is unaffected, whether Mre11 excises the DNA ends, is controlled by the protein C1QBP. C1QBP binds to Mre11 directly to form the MRC (Mre11, Rad50, and C1QBP) complex to stabilize Mre11 and limit its nuclease activity [13].

In *Saccharomyces cerevisiae*, the Mre11 protein contains two functional domains, a nuclease domain in the N-terminus required for DSB repair, and the dsDNA-binding domain in the C-terminus essential for its meiotic functions such as DSB formation and chromatin modification [45]. In this study, we found that the OsMre11 protein also contains two functional domains (Figure S1), a metallophos phosphoesterase domain near the N-terminus and a DNA-binding domain close to the C-terminus. Phosphoesterase domains are usually associated with DNA polymerases, while DNA-binding domains can promote the implementation of nuclease activity [46]. 

The first Mre11 homolog was found in the screening of meiotic mutants of *S. cerevisiae*. The mutation of *Mre11* (also known as *rad32*) in fission yeast leads to increased chromosome loss, indicating that *Mre11* plays an important role in maintaining the stability of chromosomes [31]. In *Caenorhabditis elegans*, the germ cells of the *mre-11* mutants with the treatment of γ-irradiation generated chromosomal abnormality during meiotic prophase I, indicating that the chromosomal damage repair in these germ cells was defective [32]. In humans, mutation of *Mre11* can cause ataxia-type telangiectasia-like disease (ATLD), which is characterized by chromosomal instability, radiation hypersensitivity, neurological deficits, and susceptibility to certain types of cancer [47]. When the N117S mutation occurs in *hMre11*, the interaction between Mre11 and Nbs1 in most ATLD patients weakens, the telomere length becomes unstable, and ATM cannot be recruited to repair the damaged DNA [48]. In *Arabidopsis*, the T-DNA inserted mutant *mre11-1* was abnormally developed, with dwarf plants, small siliques, few fertile seeds, and high sensitivity to damage agents. The telomere length of the mutant was increased, and the C-terminal domain of the Mre11 protein was closely related to telomere maintenance [49]. In our study, the *OsMre11* gene is widely expressed in various tissues and organs of rice (Figure 1), implying that it may function in various stages of plant growth and development. In the *mre11* of rice, vegetative growth and reproductive development were both defective, with dwarf plants and completely aborted seeds (Figure 2). The results indicate that OsMre11 plays essential roles in plant growth and development of rice. 

### 3.2. OsMre11 Is Essential for Rice Mitosis

Cell division plays an essential role in plant growth and development. In *Arabidopsis*-related research, cell division was reduced, the primary root became shorter, and the length of the RAM decreased significantly in the *nse1-1*, *nse3-1*, and *rfc4* partially complemented mutant. The *NSE1* and *NSE3* genes can promote DNA damage repair during mitosis. The *RFC4* that is involved in DNA replication and mitosis [50,51]. The full-length of the Mre11 protein in *Arabidopsis* is necessary for cell cycle arrest and transcriptional regulation. When the *Mre11* was mutated, the expression of cell cycle-related genes and transcription-related genes changed significantly [52]. In rice, the mitotic cell cycle of the *alr* mutant was abnormal, leading to delayed plant growth and decreased cell number. Further research showed that the transition of the cell cycle from G1/S to G2/M was blocked in the *alr* mutant. The phenotypic characteristics of the *alr* mutant are albinic leaves, dwarf stature, and necrotic lesions [53]. 

In this study, we found that the root length of rice *mre11* was shorter, the cell number was lower, and the cell size was smaller in *mre11* RAM than WT through a root tip transparent and staining assay (Figure 4). Further study showed that mitotic division was aberrant in the RAM of the *mre11* seedlings (Figure 5). The appearance of chromosomal fragments and chromosomal bridges in the *mre11* RAM of rice caused abnormal mitotic divisions. The ratio of EdU-labeled cells in the RAM of the *mre11* mutants were significantly lower than those in WT. Treatment with a replication inhibitor also showed that *Mre11* was very sensitive to the replication inhibitor HU (Figure 6), but not sensitive to aphidicolin (Figure S4a,b), indicating that *Mre11* responds differently to various replication inhibitors. HU is a reversible ribonucleotide reductase inhibitor, and the treatment with HU depletes nucleotides and prevents cells from entering S phase [54]. Aphidicolin can directly inhibit DNA polymerase activity, causing cells to stall at S phase [55]. Due to the different targets treated by different reagents, the sensitivity of *Mre11* to the reagents may also be different. Furthermore, we found that the expression of genes involved in DNA replication and the cell cycle decreased significantly in the *mre11* mutants (Figure 9b), indicating that *Mre11* may participate in the process of mitosis by affecting the expression of genes related to replication and the cell cycle. The changes in the expression of these genes may be caused by developmental defects resulting from the mutation of the *Mre11* gene. It may tell us that the abnormal DNA replication causes the disorder of mitosis, and fewer cell numbers, resulting in stunted plant growth. Therefore, *Mre11* has an essential role in DNA replication during the process of the mitotic cell cycle.

When mitosis is disordered, chromosome are unstable, and DNA damage occurs in the cell. Then, repair proteins must repair the damage. In addition, chromosome abnormality can also cause the cell cycle arrest. After the repair is completed, the cell cycle continues [56,57]. As an important member of damage repair, MRN responds to DNA damage in different species. In *S. cerevisiae*, the GFP-labeled repair proteins as florescent markers were used to label the location of chromosomes in living cells. If the MRX complex congregated in large numbers at the end of severely damaged chromosomes, it indicated that the complex is able to generate a response to the DNA damage [58,59,60]. In humans, targeted genome editing was performed in HEK293 cells, and the activation of DNA repair mechanisms, including MRN, could be induced [61]. In *Arabidopsis*, γ-irradiation caused the production of DSBs, which led to MRN-dependent activation of ATM and ATR kinases [12].

In our study, when WT plants were treated with DNA damage reagent MMS, the *Mre11* gene was strongly up-regulated (Figure 7), but when plants were treated with MMC, there was almost no *Mre11* response (Figure S4c,d). MMS is a DNA damaging agent and mainly acts on the N7 and N3 deoxyguanosines of DNA molecules, resulting in the formation of DNA double strand breaks (DSBs) [62,63]. MMC is a DNA cross-linking agent that can cause single or double strand breaks [63]. These results suggest that the sensitivity of *Mre11* to various damage reagents is different according to the manner of injury. The comet assay showed that DNA damage in the *mre11* mutant was much higher than in WT (Figure 7), and the gene expression related to DNA damage repair was changed significantly compared to WT (Figure 9b). Among those genes, it was reported that *Rad51* and *Mus81* were associated with HR [22,23,64,65], *ATR* responded to halt cell cycle progression and DNA damage repair [66], and *XLF* was involved in NHEJ [67]. These results illustrate that OsMre11 plays a role in the process of DNA damage repair, and may participate in both HR and NHEJ repair pathways. Through BiFC and Co-IP experiments, we detected the interactive relationship among OsMre11, OsRad50, and OsNbs1. OsMre11 could form a complex with OsRad50 and OsNbs1 (Figure S6 and Figure 8), suggesting that they may function in damage repair as a complex. Given the abnormal DNA replication in the *mre11* mutant of rice, we speculated that the emergence of DNA replication problems causes cells to recruit repair proteins including Mre11 to survive better. However, due to the deletion of the repair protein Mre11 in the *mre11* mutant, the accumulation of damaged genomes was caused, further inhibiting DNA replication.

## 4. Conclusions

The different functions of *Mre11* in many species and in meiosis have been investigated. In plant-related research, such as in *Arabidopsis*, the mutation of *Mre11* led to abnormal meiosis, cell cycle stagnation, chromosome instability, and failure of DNA damage repair [12,28,52,68]. In rice, *Mre11* was required for homologous synapsis and DNA damage repair in meiosis [35], but the role in mitosis was little known. Here, we demonstrate that *OsMre11* is indispensable for the growth and development of rice. Loss-of-function of *OsMre11* causes abnormal development of male and female gametophytes and finally leads to complete abortion of seeds. Abnormal mitosis leads to a decrease in the number of cells, which ultimately leads to the dwarf plants. Furthermore, the mutation of *OsMre11* could affect DNA replication and DSB repair, and the transcriptional response of *OsMre11* showed high sensitivity to the replication inhibitor HU and the damage agent MMS. OsMre11 could form a complex with OsRad50 and OsNbs1, and may function in the mitotic cell cycle of rice via two pathways: HR and NHEJ.

OsMre11 is essential for cell division and plays an important role in DNA replication and damage repair during the mitotic cell cycle. This study can help us better understand the molecular mechanism of *Mre11* during DNA replication and repair in rice.

## 5. Materials and Methods

### 5.1. Plant Materials and Growth Conditions

In this study, *Oryza sativa* japonica Dongjin was used as the wild type plant. The T-DNA inserted mutant *mre11* was obtained from a RISD-DB Mutant Library (Rice T-DNA Insertion Sequence Database, http://cbi.khu.ac.kr/RISD_DB.html). All plants were cultivated in a greenhouse at Wuhan University at 28–30 °C with a 14 h light and 10 h dark cycle from October to April, and in potted soil under natural conditions the rest of the year. We used the three-primer method to identify the *mre11* mutant plants, and used a pair of primers on the genome to amplify the wild-type band, and a pair of primers on the genome and T-DNA to amplify the mutant band.

A 3016 bp upstream DNA fragment before the initiation codon of *OsMre11* as the 5′-UTR, all nucleotide sequences (5184 bp) of the open reading frame, and the 2361 bp fragment after the stop codon as the 3′-UTR were used as the full-length sequence information for constructing the complementary vector. Then, this entire fragment was ligated to the *pCAMBIA2301* vector for genetic transformation. The background of complementary transgenic plants was *mre11*. In addition, *Osmre11-cr* transgenic plants were obtained via CRISPR/Cas9 (clustered regularly interspaced short palindromic repeats/Cas9) technique [69]. The background of all CRISPR transgenic plants was rice variety Dongjin.

### 5.2. Phylogenetic Analysis

*OsMre11* (LOC_Os04g54340) is a single copy gene in *Oryza sativa*. The OsMre11 protein shares a relatively conserved domain with its orthologs in other species. Its orthologs were obtained from the NCBI database (National Center for Biotechnology Information) (https://www.ncbi.nlm.nih.gov/). The phylogenetic tree was constructed using ClustalIX 1.83 to load the relevant sequence, and MEGA 5.1 to convert the file format to the object document based on the neighbor-joining method (bootstrap = 1000).

The accession numbers of proteins discussed in this article are from the National Center for Biotechnology Information (NCBI), Mre11 (*Oryza sativa*)- AY935255.1, Mre11 (*Arabidopsis thaliana*)-OAO95209.1, Mre11 (*Homo sapiens*)-NP_001317276.1, Mre11 (*Vitis vinifera*)-XP_010644252.1, Mre11 (*Mus musculus*)-NP_001297657.1, Mre11 (*Populus euphratica*)- XP_011044534.1, Mre11 (*Zea mays*)-ACG43091.1, Mre11 (*Sorghum bicolor*)- XP_021321224.1, Mre11 (*Caenorhabditis elegans*)-NP_505736.2, Mre11 (*Drosophila melanogaster*)-AAD33591.1, Mre11 (*Danio rerio*)- NP_001307188.1, Mre11 (*Saccharomyces cerevisiae*)-BAA02017.1, Mre11 (*Glycine max*)-XP_003539581.1, Mre11 (*Nicotiana tabacum*)-XP_016437115.1, Mre11 (*Triticum aestivum*)-SPT20634.1, and Mre11 (*Escherichia coli*)- AZM66280.1.

### 5.3. qRT-PCR Assay

Total RNAs of vegetative and reproductive organs were extracted using TRIzol ^®^ reagent. qRT-PCR (quantitative real-time polymerase chain reaction) was performed using SYBR green fluorescence with a CFX Connect^TM^ real time system (Bio-Rad) [70]. *Actin* was used as the housekeeping gene to normalize the expression of genes in various RNA samples. Three independent biological replicates and three technical replicates were included during final data analysis. In the tests, all primers are listed in Table S1.

### 5.4. GUS Staining

A 3016 bp upstream DNA fragment before the initiation codon of *OsMre11* gene was amplified as the promoter. This promoter was then ligated to the *pCAMBIA-1381×b* vector to construct pMre11::GUS, and invaded into rice Dongjin callus through agrobacterium-mediated transformation [71]. Different tissues and organs from transgenic positive plants were obtained for GUS staining analysis. After staining with GUS dye (0.1 mol/L Na_3_PO4, 10 mmol/L Na_2_EDTA, 0.5 mmol/L K_4_[Fe(CN)6], 0.5 mmol/L K_3_[Fe(CN)6], 0.5 mg/mL X-Gluc, 0.5% Triton-X100), and decolorizing with 75% and 100% ethanol, the samples were photographed with a Canon EOS 70D digital camera. 

### 5.5. Subcellular Localization of OsMre11

The full-length CDS (without stop codons) of *OsMre11* was cloned into *35S-GFP* (transformed from *mpCAMBIA1300*) vector to generate the *35S::OsMre11-GFP*. The plasmid was transformed into agrobacterium GV3101, and then injected into tobacco (*Nicotiana benthamiana*) leaf epidermis cells and incubated for 36 h. The signal of fluorescent proteins was visualized under a Leica SP8 confocal laser-scanning microscope. The excitation and emission wavelengths for green fluorescent protein (GFP) are 488 and 507 nm, respectively.

### 5.6. Plant Phenotype Analysis

The rice roots and ovaries were fixed with FAA (formaldehyde:acetic acid:50% ethyl alcohol = 5:6:89) for at least 24 h, and rehydrated in a series gradient of alcohol. Then, the roots were dyed with eosin B and dehydrated in alcohol, transparent in the methyl salicylate, and photographed under a Leica SP8 confocal microscope. 

Root meristem size was determined by measuring the length from the quiescent center to the first elongated cell in the 4th cortex layer. When the length of a cortex cell was twice that of the neighbor cell, it was considered to be the first elongated cortex cell. The average cell number of the root meristem was quantified in the 4th cortex layer [72]. The average cell size of the root meristem was the length of the root meristem divided by the average cell number of the root meristem. 

### 5.7. Mitosis Assay

Rice roots were fixed in 4% paraformaldehyde for 1 h at room temperature, and washed three times for 5 min each time. Then, the roots were digested in 0.5% cellulase R-10 and 0.5% macerozyme R-10 for 1 h, and washed three times again. The root meristem was isolated and mounted on a glass slide. After being dyed with DAPI (4′,6-diamidino-2-phenylindole), the samples were pressed lightly, and their mitotic stages were recorded under an Olympus FluoView FV1000 confocal microscope.

### 5.8. Treatment with DNA Replication Inhibitors and DNA Damage Reagents

The seeds of rice Dongjin were germinated on 1/2 MS medium for 10 days. The germinated seedlings that were selected for treatment tests grew well. Treatment reagents were HU (0, 8 and 16 mM), aphidicolin (0, 50, 100, and 150 µM), MMS (0, 272, 544, and 816 µM), and MMC (0, 30, 60, 90, and 150 µM). Samples treated at 0.5, 1, and 2 days were collected. Three seedlings were collected as one biological sample. Three independent biological replicates and three technical replicates were included in final data analysis.

### 5.9. EdU Staining Analysis

The 7-day-old seedlings were prepared for the EdU (ethynyl deoxyuridine) staining experiment [51]. The root tips were incubated in 50 µM EdU solution for 2.5 h, fixed in 4% paraformaldehyde and treated with Apollo reagent according to the cell-light EdU Apollo 488 in vitro imaging kit (RiboBio). The fluorescence was detected under a Leica SP8 confocal microscope and photographed.

### 5.10. Comet Assay

The nuclei of root apical meristem cells from 7-day-old rice seedlings were mixed with low melting-point agarose, and then the mixture was tiled on normal melting-point agarose on a glass slide. After the mixture was unwound and subjected to electrophoresis according to the protocol described previously [50], the mixture was dyed with PI (propidium iodide), and observed under a Leica SP8 confocal microscope. The data were analyzed using CometScore software (http://www.autocomet.com). DNA damage was calculated by averaging the values of the percentage of DNA in tails from more than 70 comets.

### 5.11. Homology Modeling

The sequence of OsMre11, OsRad50, OsNbs1, and their homologous proteins were downloaded from NCBI. Their 3D structures were modeled using the SWISS-MODEL server (http://swissmodel.expasy.org/) [73,74]. The homology templates were 4yke.1.A, 5dac.1.A, and 3hue.1.A, respectively [75]. The PDB files of the modeled proteins were displayed and downloaded using Swiss-Pdb Viewer.

### 5.12. BiFC Assay

The full length ORFs (without stop codons) of *OsMre11* (LOC_Os04g54340), *OsRad50* (LOC_Os02g29464), and *OsNbs1* (LOC_Os10g34580) were inserted into the *pCAMBIA-SPYNE* and *pCAMBIA-SPYCE* vectors, respectively, to make different plasmids. Then, the plasmids were injected into tobacco (*Nicotiana benthamiana*) leaf epidermis cells for BiFC (bimolecular fluorescence complementation) assay. Approximately 36 h later, fluorescence signals were observed. YN and YC stand for *pCAMBIA-SPYNE* and *pCAMBIA-SPYCE* empty vectors in the text respectively. The detailed procedure was performed according to Sparkes’ method [76].

### 5.13. Co-IP Analysis

For Co-IP (co-immunoprecipitation) analysis, the CDS (without stop codons) of OsRad50 and OsNbs1 were obtained to construct the 35S::OsRad50-3×Flag and 35S::OsNbs1-3×Myc vectors, respectively. The 35S::OsMre11-GFP, 35S::OsRad50-3×Flag, and 35S::OsNbs1-3×Myc vectors were injected into tobacco leaf epidermis cells and cultivated for 36 h. The proteins used for the Co-IP assay were incubated with anti-Myc and anti-Flag mouse antibodies, respectively. Immunoblotting was performed with anti-GFP, anti-Flag and anti-Myc rabbit antibodies, respectively. The detailed process was performed as described previously [77].

### 5.14. RNA-Seq Assay

The 7-day-old seedlings of WT and *osmre11* were harvested. Three plants were used as one sample, and both of WT and the *osmre11* contained two samples. Their whole RNA was extracted and sequenced by GENEWIZ Company. The steps of RNA extraction are briefly described below. The samples were lysed with Trizol reagent. RNA was extracted with chloroform and precipitated with isopropanol. In addition, 75% alcohol was used to clean the impurities, and finally RNA was dissolved with RNase-free water. Sequencing reads were mapped to the reference genome and transcriptome sequences (http://rice.plantbiology.msu.edu/pub/data/Eukaryotic_Projects/o_sativa/annotation_dbs/pseudomolecules/version_7.0/). The model of NGS was Illumina HiSeq2000. The transcriptome sequencing experiment process includes RNA extraction, RNA sample quality detection, library construction, library purification, library detection, library quantification, sequencing cluster generation, and computer sequencing. The length we obtained was 150 bp, and the numbers of reads were 49,948,012, 43,922,662, 43,510,058, and 52,841,196. Htseq software (V 0.6.1) was used for gene expression calculation, DESeq2 (V1.6.3) of Bioconductor software was used for gene difference analysis, and DEXSeq (V1.18.4) software was used for differential exon usage analysis. The threshold for significance was *p* < 0.05. GO (Gene ontology) analysis was carried out by WEGO (Web Gene Ontology Annotation Plot; http://wego.genomics.org.cn/cgi-bin/wego/index.pl). KEGG (Kyoto Encyclopedia of Genes and Genomes) was analyzed by the method described previously [78]. From KEGG correlation analysis results, homologous recombination and non-homologous end joining repair pathways were selected, and the related changed genes were displayed [78,79,80]. All the raw data of RNA-seq was uploaded in the GEO database of NCBI. The GEO series is GSE163616. Gene Expression Omnibus (https://www.ncbi.nlm.nih.gov/geo/query/acc.cgi?acc=GSE163616).

## Data Availability

The T-DNA inserted mutant *mre11* was obtained from a RISD-DB Mutant Library (Rice T-DNA Insertion Sequence Database, http://cbi.khu.ac.kr/RISD_DB.html).

## Supplementary Materials

The following are available online at https://www.mdpi.com/1422-0067/22/1/169/s1, Figure S1: Comparison of the amino acid sequences of OsMre11 and its homologs. Figure S2: Phylogenetic analysis of OsMre11 and other 11 homologs. Figure S3: Expression patterns of OsRad50 and OsNbs1 in rice. Figure S4: Relative expression level of OsMre11 responded to Aphidicolin and MMC in 10DAG seedlings. Figure S5: The three-dimensional structure of Mre11, Rad50, and Nbs1 in various species. Figure S6: BiFC assay shows the interaction relationships between OsMre11, OsRad50, and Os-Nbs1 in tobacco leaf epidermis cells. Figure S7: RNA-seq analysis of wild type and the mre11 mutant in rice. Table S1: Primers (5′ to 3′) used in the experiments.
