# Supplementary material for: OsMre11 Is Required for Mitosis during Rice Growth and Development
Source: Int J Mol Sci. 2020 Dec 26;22(1):169. doi: 10.3390/ijms22010169 (PMC7795355; doi:10.3390/ijms22010169)
Supplement: Supplementary file 1 [file ijms-22-00169-s001.pdf]

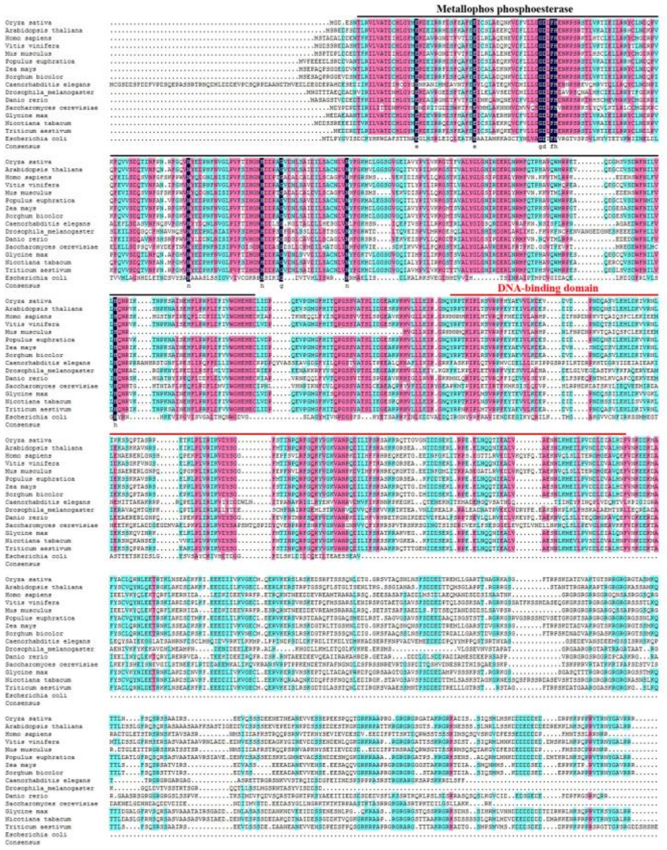

Figure S1. Comparison of the amino acid sequences of OsMre11 and its homologs. The black lines label metallophos phosphoesterase domain, while the red lines represent DNA-binding domain.

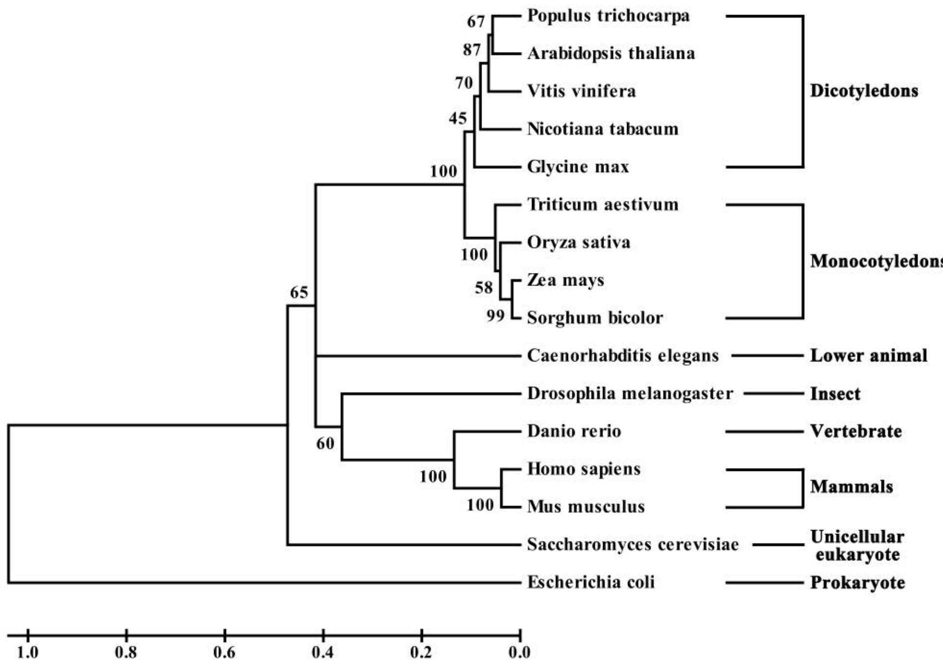

Figure S2. Phylogenetic analysis of OsMre11 and other 11 homologs. The scale bar represents the number of amino acid substitutions per site.

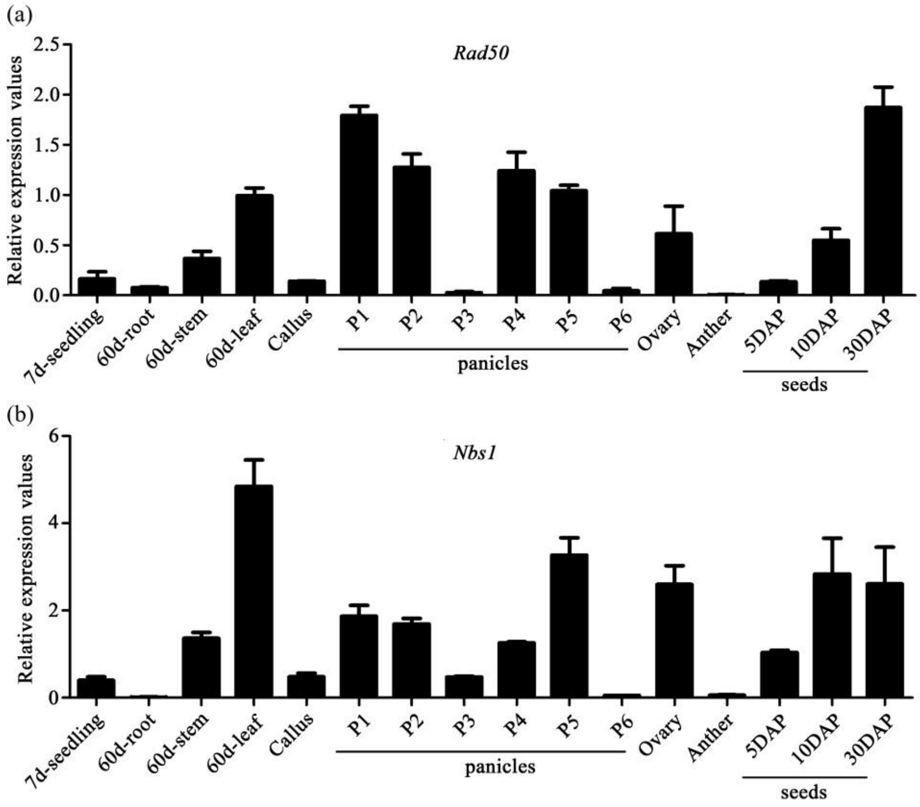

Figure S3. Expression patterns of *OsRad50* and *OsNbs1* in rice.Expression values of *OsRad50* (a) and *OsNbs1* (b) in 7-day-old seedling, 60-day-old root, 60-day-old stem, 60-day-old leaf, callus, panicles (P1: 0-3cm spikelet; P2: 3-5cm spikelet; P3: 5-10cm spikelet; P4: 10-15cm spikelet; P5: 15-22cm spikelet; P6: 22-30cm spikelet), ovary, anther and seeds (5DAP: 5days after pollination; 10DAP: 10 days after pollination; 30DAP: 30 days after pollination).

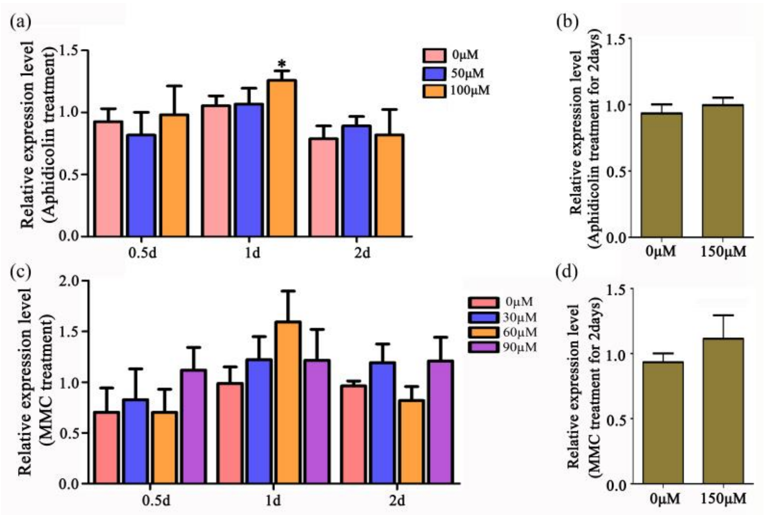

Figure S4. Relative expression level of *OsMre11* responded to Aphidicolin and MMC in 10DAG seedlings. (a) The concentration of Aphidicolin is 0μM, 50μM and 100μM. The materials with the treatment of 0.5, 1 and 2 days were collected. (b) The concentration of Aphidicolin is 150μM, and the material with the treatment of 2 days was collected. (c) The concentration of MMC is 0μM, 30μM, 60μM and 90μM, respectively. The materials with the treatment of 0.5, 1 and 2 days were collected. (d) The concentration of MMC is 150μM, and the material with the treatment of 2 days was collected. The one asterisk represents a statistically significant difference according to Student's t-test (*, $p < 0.05$).

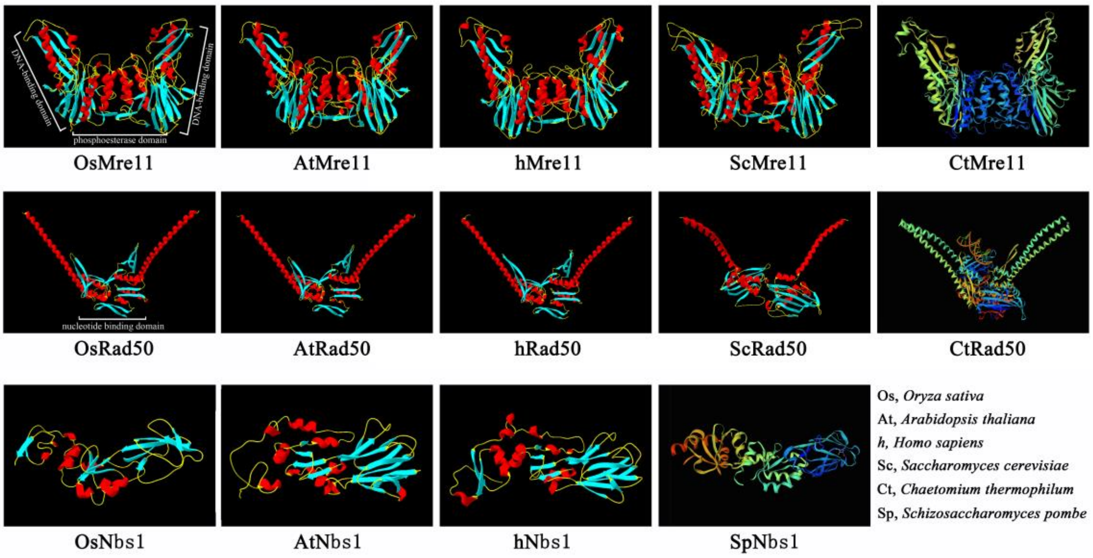

Figure S5. The three-dimensional structure of Mre11, Rad50, and Nbs1 in various species. OsMre11, AtMre11, hMre11, and ScMre11 are modeled according to the crystal structure of its homologue CtMre11 through SWISS-MODEL (https://www.swissmodel.expasy.org/). OsRad50, AtRad50, hRad50, and ScRad50 are modeled according to the crystal structure of its homologue CtRad50. OsNbs1, AtNbs1, and hNbs1 are modeled according to the crystal structure of its homologue SpNbs1.

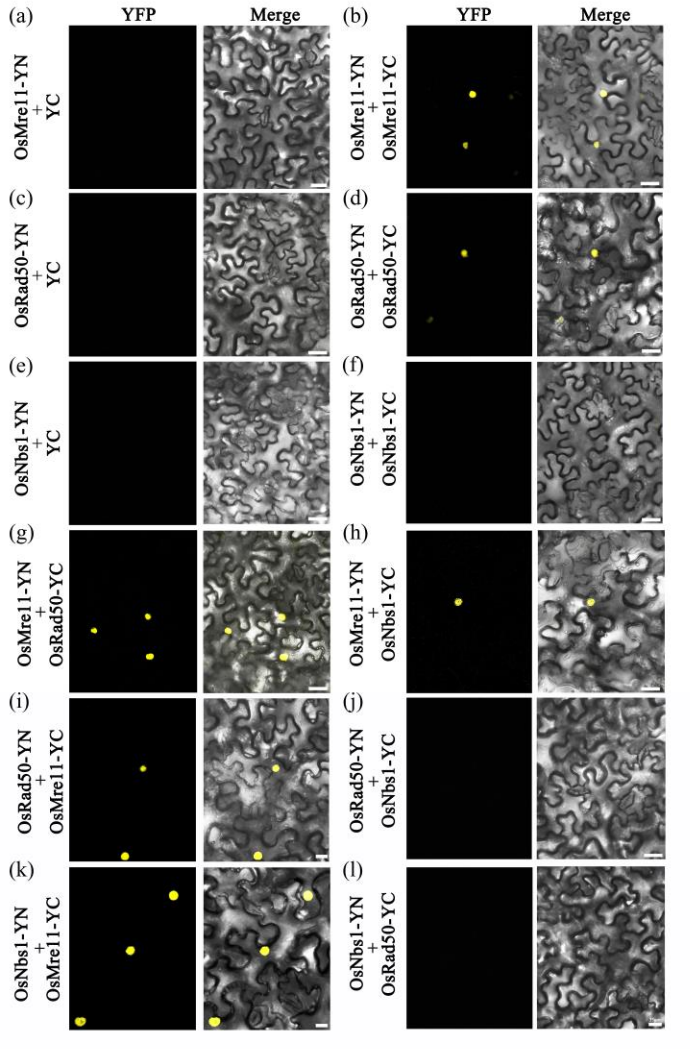

Figure S6. BiFC assay shows the interaction relationships between OsMre11, OsRad50, and OsNbs1 in tobacco leaf epidermis cells. (a, c, e) OsMre11-YN+YC, OsRad50-YN+YC and OsNbs1-YN+YC were the control groups. YC is an empty carrier. YN and YC stand for *pCAMBIA-SPYNE* and *pCAMBIA-SPYCE* empty vectors respectively. (a-l) The epidermal cells were observed at 36 hours after being co-transformed. Scale bars represent 50μm.

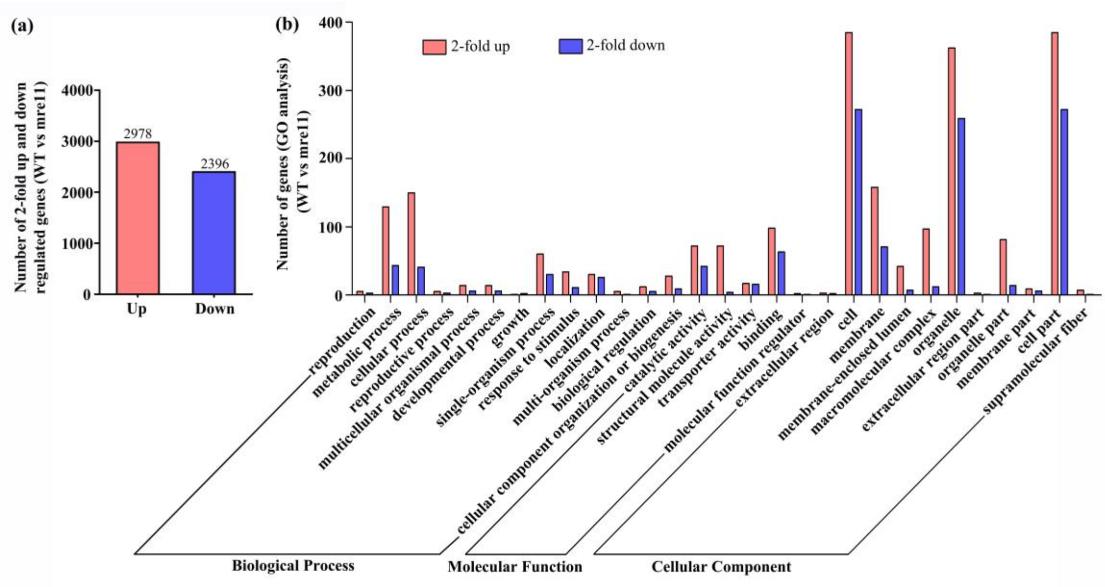

Figure S7. RNA-seq analysis of wild type and the *mre11* mutant in rice. (a) Number of 2-fold up and down regulated genes (WT vs *mre11*). (b) Histogram presentation of gene ontology (GO) classification based on RNA-seq data. The red histograms show up-regulated genes (2-fold up), while the blue histograms represent down-regulated genes (2-fold down).

Table S1. Primers (5′ to 3′) used in the experiments.

Primers for Mutant Verification

	FP	RP
mre11	ACAA-GATGGCGTTTTATGCC	AGTTCACCAGGTCATTTGCC
2715LB	GTCTAAGCGTCAATTTGTT	

Primers for complementation

	FP	RP
Os-Mre11-5′UTR	CGCGGATCCTCCAAC-GGGCTCACCAACT	GTGTTGCTTTCGTCTCCCATT-GCCGGTGTTGGTTCAGCTT
OsMre11-CDS	AAGCTGAACCAACAC-CGGCAATGGGAGAC-GAAAGCAACAC	AGACTTAGACTAACAA-GAGGTCATCTCCTCCTAACAGCTC
Os-Mre11-3′UTR	GAGCTGTTAGGAGGA-GATGACCTCTTGTTAG-TCTAAGTCT	CGCGTCGACCGAA-GATCCCTCGCAACTA
pCAMBIA2301		CCTCTTCGCTATTACGC

Primers for qRT-PCR

	FP	RP
Actin	CCCCAAGGCCAATCGTGAG	ACGCCCAGCAAGGTCGAGA

OsMre11	AAAGATGCTACCGATGT	CATTAGTCTCATTTTCC
OsRad50	ATGAGCCGACCACCA	ATGAGCCGACCACCA
OsNbs1	CTCGGAAGTGGAGTGAT	GAAGGCATTTCGGTGT
MCM3	GGGACTATGGTCTGCG	GCCTACGAAAGATGTGAT
MCM4	GCTGCTTGGTTTGCG	TGGAGTGCCACCTTCTG
MCM5	TGGGAAAGGTTCATCAGCA	ACTCCACCATCAGCCAAAA
MCM6	AAAAGCAGGAATACAAGC	TGATGGACCCTCACAAT
MCM7	AGACTGTTAG-TATTGCGAAGG	CTGGAGGTAGATTGATGTTTT
ORC5	CGCCCTCTTCGCTTCT	GCTGACCACCCTTATCCC
RAD9	GGAA-GAGCCTCCTGATGTTG	GTAGTGCGGTGTTGTTTGG
cyclin-A3-2	CAGGAGGTTGTCAA-GATGGA	GGAGACAGCCGTAGTCAAGTAG
cyclin-B2-2	AATGGAGGGCGTCAAG	TTAGCGGCAGGTTTATC
cyclin-D3-1	TTCTTGGGTTGTTGGG	GATGTGCTGCTGCTCC
cyclin-D6-1	CTCGCCTTCCTCGGCTTCTT	AACTCCGCCATCTTCACCTCC
ATM	ACTTGTTGCCTTCGTAA	AAGTGGCTCCAAATCTC
ATR	CCTAAGAATGGACCCG	AGGCAGCAGAAACAAAT
Rad51	TGCGAGCCAACTTCAT	AGAGCCAGTTTCTATCCC
Mus81	CAGGAGGGTCAAAGC	GTGGCGTCAATAAGC
Ku70	GAGGGAGATGAAA-TAGTGG	AGTTGAACGGATTAGCG
Ku80	TCACTCCGAATCCCA	GCTCAAATACATTGCCTA
XLF	CAAGCACTGGGAATG	TGGAAGCGGAACTG

Primers for CRISPR/Cas9

	FP	RP
mre11-cr	GGCAC-TCTGAATTTCAGCCAATCA	AAACTGATTGGCTGAAATTCAGAG
Ubi-RP		GATAAACTGCACTTCAAACA

Primers for subcellular localization fusion constructs

	FP	RP
Mre11-GFP	TGCTCTAGATCCAAC-GGGCTCACCAACTC	CGCGGATCCTGCCGGTGTT-GGTTCAGCTT
35S-GFP-RP		CGCACAATCCCACTATCCTTCG
pCAMBIA1300	CGGGCCTCTTCGCTATTACG	AGGCACCCCAGGCTTTACACT

Primers for tissue expression

	FP	RP
Mre11-GUS	CGCGGATCCTCCAAC-GGGCTCACCAACTC	CGCGTCGACCATTGCCGGTGTT-GGTTCAGCTT
GUS-RP		AACGCTGATCAATTCCACAG

Primers for BiFC

	FP	RP
OsMre11	TGCTCTAGAATGGGA-	TCCCCCGGGTCTCCTCCTAACAG

	GACGAAAGCAACAC	CTCCGT
OsRad50	CGGACTAGTATGAGCAC-GGTGGACAAGAT	TCCCCCGGGGTCAAA-GATCTCCTGGGCTT
OsNbs1	TGCTCTA-GAATGGTGTGGGCGCTGACCCC	TCCCCCGGGTCTGCGGCCGG-TAAGCATGG
NosT		GCAAGACCGGCAACAGGATTCA

Primers for Co-IP

	FP	RP
Rad50-3×Flag-1	CACGGTCTCG-GATCCATGAGCACGGTG-GACAAGAT	CTT-GTCATCATCGTCCTTATAGTCCTTATCGTCGTCATCCTT-GTAATCGTCAAA-GATCTCCTGGGCTT
Rad50-3×Flag-2		CGCGTCGACTCATTTATCGTCATCATCTTTGTAGTCCTT-GTCATCATCGTCCTTATAGTC
Nbs1-3×Myc-1	TGCTCTA-GAATGGTGTGGGCGCTGACCCC	CTCCTCAGAAATAAGTTTTT-GCTCAA-GATCCTCCTCAGAAATCAACTTTTGCTCTCTGCGGCCGGTAA-GCATGG
Nbs1-3×Myc-2		CACGGTCTCGGATCCTCAC-TAC-AAATCTTCTTCAGAAATCAATTTTTGTTCAAGATCCTCCTCAGAAA-TAAGTTTTTGC
